# High-fiber diet ameliorates gut microbiota, serum metabolism and emotional mood in type 2 diabetes patients

**DOI:** 10.3389/fcimb.2023.1069954

**Published:** 2023-01-30

**Authors:** Lihua Chen, Bo Liu, Lixia Ren, Hao Du, Chunhua Fei, Chang Qian, Bin Li, Ruixia Zhang, Haixia Liu, Zongjie Li, Zhiyong Ma

**Affiliations:** ^1^ School of Perfume and Aroma Technology, Shanghai Institute of Technology, Shanghai, China; ^2^ Department of Gastroenterology, Mental Health Center of Fengxian District, Shanghai, China; ^3^ Shanghai Ninth People’s Hospital, Shanghai Jiao Tong University School of Medicine, Shanghai, China; ^4^ Safety & Quality Management Department, Sino-science Yikang (Beijing) Biotech Co., Ltd, Beijing, China; ^5^ Shanghai Veterinary Research Institute, Chinese Academy of Agricultural Science, Shanghai, China

**Keywords:** dietary fiber, gut microbiota, serum metabolome, depression, anxiety, type 2 diabetes mellitus

## Abstract

Previous studies have demonstrated that patients with type 2 diabetes mellitus (T2DM) often had the problems of fecal microbiota dysbiosis, and were usually accompanied with psychiatric comorbidities (such as depression and anxiety). Here, we conducted a randomized clinical study to analyze the changes in gut microbiota, serum metabolism and emotional mood of patients with T2DM after consumption of a high-fiber diet. The glucose homeostasis of participants with T2DM was improved by the high-fiber diet, and the serum metabolome, systemic inflammation and psychiatric comorbidities were also altered. The increased abundances of *Lactobacillus*, *Bifidobacterium* and *Akkermansias* revealed that the proportions of beneficial gut microbes were enriched by the high-fiber diet, while the abundances of *Desulfovibrio*, *Klebsiella* and other opportunistic pathogens were decreased. Therefore, the current study demonstrated that the intestinal microbiota alterations which were influenced by the high-fiber diet could improve the serum metabolism and emotional mood of patients with T2DM.

## Introduction

Accumulating studies have demonstrated that there were intimate correlations between type 2 diabetes mellitus (T2DM) and gut microbiota. By modulating the structure of gut microbiota, a specially designed high-fiber diet was proved to be able to provide obvious beneficial effects for patients with T2DM (Zhao et al., 2018). In fact, the modulation of gut microbiota could not only increase the relative abundances of *Lactobacillus, Bifidobacterium* and other beneficial microbes, and could also improve the plasma and the fecal bile acid metabolism of patients with T2DM (Gu et al., 2017). Metagenome-wide association study in a cohort of young Chinese individuals identified an obesity-associated gut microbial species (*Bacteroides thetaiotaomicron*), which could alleviate diet-induced body-weight gain and adiposity by reducing plasma glutamate concentrations (Liu et al., 2017). These findings indicated that the intestinal microbiota might play a critical role in treating obesity and T2DM. Several genera of beneficial bacteria (such as *Allobaculum, Bacteroides, Blautia, Butyricoccus*, and *Phascolarctobacterium*) with short-chain fatty acid (SCFA)-producing properties were proved to be associated with the prevention of obesity and insulin resistance in high-fat diet (HFD) fed rats (Zhang et al., 2012; Zhang et al., 2015). A large structural survey of fecal microbiota in 314 healthy Chinese young adults revealed that nine genera of bacteria (including *Phascolarctobacterium, Roseburia, Blautia, Faecalibacterium, Clostridium, Subdoligranulum, Ruminococcus, Coprococcus* and *Bacteroides*) with SCFA-producing properties were certified to be necessary for maintaining the host’s health (Zhang et al., 2015). Therefore, many novel strategies (such as prebiotic, probiotic and fecal microbiota transplantation) targeting the gut microbiota could be developed for type 2 diabetes interventions.

The clinical symptoms of anxiety and depression were often observed in patients with T2DM, and the reason might lie in that the diabetic nutrient restrictions and the imbalanced gut microbiota influence the patient’s emotional state (Collins et al., 2009; Semenkovich et al., 2015). The gut microbiota could influence the host’s central neurochemistry and change their stress responses and cognitive behaviors, and the communications between the gut and brain might be related to the various kinds of microbial metabolites, including catecholamines, serotonin, gamma-aminobutyric acid, and SCFAs (Ho & Ross, 2017). In particular, administration of SCFAs were proved to be able to alleviate the stress-induced responsiveness as well as the intestinal permeability, and could also influence the brain immune homeostasis, energy metabolism, and physiological states (Wouw et al., 2018; Valles-Colomer et al., 2019). Moreover, functional food consumption could obviously decrease the autoreactive T cell numbers in lymphoid tissues and decrease the serum concentrations of inflammatory cytokines (Marino et al., 2017). Therefore, the bidirectional communications between the gut and brain could be influenced by nutritional interventions.

Nowadays, personalized nutritional interventions based on transcriptomics, proteomics, and metabolomics techniques could provide additional benefits for patients with T2DM. Consumptions of functional foods and bioactive ingredients could enhance the anti-oxidant properties, the anti-inflammatory activities, the anti-cholesterol activities, and the insulin sensitivity of patients with T2DM (Alkhatib et al., 2017; Zhang and Gregg, 2017; Laksir et al., 2018). The gut microbiota could transduce the nutrients signals and retard the development of chronic inflammation and metabolic disorders, and the administration of functional foods could also increase the abundances of *Akkermansia, Bifidobacteria, Lactobacillus, Bacteroides* and *Prevotella* (Shanahan et al., 2017). When the food components of dietary fiber, polyphenols, flavonoids, sterols, and unsaturated fatty acids were digested by the gut microbes, various kinds of microbial metabolites were produced to treat the obesity, non-alcoholic fatty liver disease (NAFLD), and T2DM (Lyu et al., 2017). By regulating the homeostasis of innate and adaptive immune system, the diverse microbiota-derived bioactive molecules could regulate the health and disease of the host (Blander et al., 2017). Recent studies had also proved that the symbiotic bacteria could influence the development and function of the nervous system, which were related to anxiety, depression, and other mental diseases (Sharon et al., 2016). In fact, most of the neurotransmitters in the brain (such as 5-hydroxytryptamine and γ-aminobutyric acid) were produced by the gut microbes, therefore the microbiome-gut-grain axis had a critical impact on stress responses, depression and anxiety (Dinan and Cryan, 2017). Thus, manipulation of functional foods towards the gut microbiota could be applied to fight against the metabolic syndromes and the central nervous system diseases (Barratt et al., 2017; Maier and al'Absi, 2017).

In this study, the profiles of fecal microbiota samples from patients with T2DM altered by a high-fiber diet were studied, and the relations between the microbial communities and the host’s clinical features were also determined and analyzed.

## Materials and methods

### Subject recruitment

This study was a randomized, open-label, parallel-group clinical trial in T2DM patients with a 4-week treatment period. The study was performed according to the principles of the Declaration of Helsinki (2008), and the study protocol was approved by the Ethics Committee of Shanghai Jiao Tong University School of Medicine (ID: SNPH2017-026). All the participants signed the informed consent forms.

During the initial screening period, patients with plasma HbA1c levels (6.5%-12.0%) were recruited. Patients who had taken antibiotics, probiotics and prebiotics during the previous 3 months were excluded. Other exclusive criteria included type 1 diabetes, severe hepatic diseases, gastrointestinal surgery and severe mental illness. Patients were also excluded if they had severe organic diseases, including cancer, coronary heart disease, and cerebral apoplexy.

### Study design

After a 2-week washout period, 17 patients clinically diagnosed with T2DM enrolled in the clinical trial and were randomly assigned into two groups using the SAS software. As an open-label, parallel-group study, the control group (n = 8 patients) received usual care, including patient education and dietary recommendations based on the 2013 Chinese Diabetes Society guidelines for T2DM; the treatment group (n = 9 patients) received whole grains, prebiotics, and traditional Chinese medicinal foods composed high-fiber diet. The high-fiber diet for the treatment group consisted of several whole grains and traditional Chinese medicinal food (shown in **Table 1**).

**Table 1:** The components of the high fiber diet.

Components	Composition
Ash content (g/100g)	4.25
Moisture (g/100g)	3.56
Carbohydrate (g/100g)	63.7
Protein (g/100g)	9.47
Fat (g/100g)	1.07
Fiber (g/100g)	17.9
Soluble fiber (g/100g)	5.6
insoluble fiber (g/100g)	12.3
Vitamin E (mg/kg)	13.7
Vitamin K (μg/100g)	25.3
Vitamin B1(mg/100g)	0.52
Vitamin B2 (mg/100g)	0.21
Vitamin C (mg/100g)	0.3
Folate (μg/100g)	47.1
Sodium (mg/kg)	900
Potassium (mg/kg)	872
Magnesium (mg/kg)	630
Iron (mg/kg)	32.67
Zinc (mg/kg)	12
Manganese (mg/kg)	10
Calcium (mg/kg)	1350
Phosphorus (mg/100g)	260
Iodine (mg/kg)	0.06
Chromium (mg/kg)	1.5
molybdenum(mg/kg)	41
Linoleic acid (g/100g)	0.12
choline(mg/100g)	2.1
L-carnitine (mg/kg)	3.9
Taurine (mg/100g)	1.48
Energy (kJ/100g)	1263.66

The recruited participants received either acarbose (100 mg; 3 times/day) plus common diet for T2DM (control group) or acarbose (100 mg; 3 times/day) plus the high fiber diet (treatment group) for 8 weeks, while all the patients received the same total caloric and macronutrients prescriptions and followed the exercises advice according to the Chinese Diabetes Society.

### Anthropometric measurement and evaluation

The assessments of fasting blood glucose (FBG), glycosylated hemoglobin (HbA1c), serum insulin, C-peptides, triglyceride (TG), total cholesterol (TC), high-density lipoprotein cholesterol (HDL-c), and low-density lipoprotein cholesterol (LDL-c) were detected before and after the treatment. The serum levels of interleukin-1β (IL-1β), interleukin-6 (IL-6), monocyte chemotactic protein-1 (MCP-1), and tumor necrosis factor-α (TNF-α) were quantified by enzyme-linked immunosorbent assays (ELISAs), respectively. The depression and anxiety symptoms of all participants were evaluated by the validated questionnaires of Hamilton Anxiety Scale (HAMA) and Hamilton Depression Scale (HAMD).

### Fecal DNA extraction and high-throughput sequencing

The fecal samples were collected for gut microbiota analysis before and after the treatments. Microbial genomic DNA was extracted using an InviMagH Stool DNA kit referred to the previous study (Ling et al., 2016). The extracted DNA was then verified by agarose gel electrophoresis following the manufacturer’s instructions. The V3-4 hypervariable region of 16S rRNA genes was amplified by PCR using the barcoded-fusion primers, and then the PCR amplification products were separated using agarose gels (Chung et al., 2016; Li et al., 2016; Song et al., 2017). The PCR products from the gels without primer dimers and contaminant bands were used for Illumina MiSeq high-throughput sequencing by Personal Biotechnology Co., Ltd., Shanghai, China.

### Bioinformatics and statistical analysis

The quality control and sequence filtering of raw reads was performed according to the barcode matching and sequence overlapping using QIIME (version 1.9.1). The filtered high-quality reads were clustered into operational taxonomic units (OTUs) with a similarity level of 97%, then the obtained OTUs were used for taxonomical assignments by RDP classifier (Chen et al., 2019; Ghosh et al., 2021). The microbial diversities and taxonomic compositions of the sampled bacterial communities were implemented using the R package software (Zhang et al., 2015). The contributions of bacterial community genes for potential function were predicted using PICRUSt package through the EggNOG Database (Gao et al., 2017).

### Statistical analysis

The data statistical analyses were compared using a one-way analysis of variance (ANOVA) by the SPSS Data Analysis Program (version 17.0; SPSS Inc., Chicago, IL, USA) at the end of each bioassay. All mean comparison was performed using Fisher’s least significant difference test (LSD) with a significance level of *P* < 0.05.

## Results

### The glucose homeostasis of participants with T2DM was improved by the high-fiber diet

**Figure 1:**
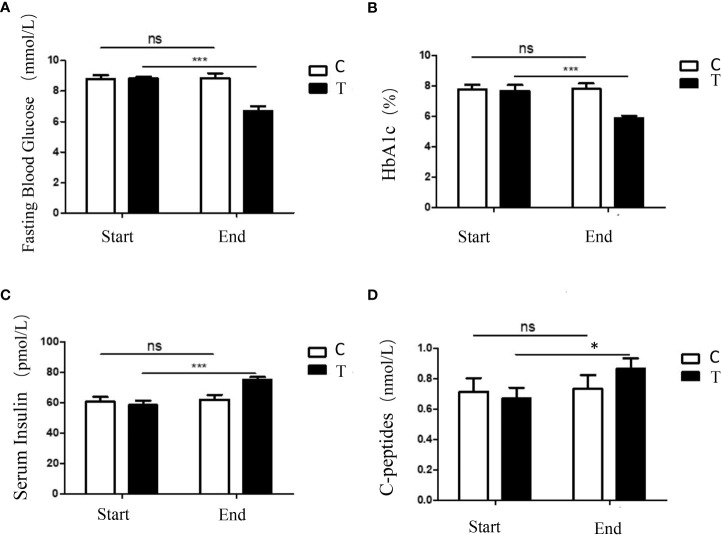
A high-fiber diet improved the glucose homeostasis in participants with T2DM. Changes in fasting blood glucose **(A)**, the percentage of HbA1c **(B)**, serum insulin **(C)**, and C-peptides **(D)** of the participants during the intervention were shown. The symbols * indicates p< 0.05, symbols*** indicates p< 0.001, ns indicates no significant difference.

After 8 weeks of intervention, the glucose homeostasis of the treatment group was significantly improved by the high-fiber diet. Compared with the control group, the HbA1c levels and FBG levels of the treatment group decreased significantly, while the levels of serum insulin and C-peptides of the treatment group increased significantly (**Figure 1**). These results demonstrated that consumption of a sufficient amount of fermentable fiber could provide obvious metabolic benefits for patients with T2DM.

### Changes in the serum lipid metabolism profiles

To observe the blood lipid profiles influenced by the high-fiber diet, the serum levels of TC, TG, LDL-C and HDL-C were measured and analyzed. Compared with the control group, the serum concentrations of TC, TG and LDL-C in the treatment group were decreased, while the serum levels of HDL-C in the treatment group were increased (shown in **Table 2**).

**Table 2:** Changes of the serum lipid metabolism parameters.

Item	Start		End	
	C	T	C	T
TC, mmol/L	5.16 ± 0.61	5.12 ± 0.57	5.21 ± 0.44	4.2 ± 0.49**
TG, mmol/L	2.72 ± 0.34	2.78 ± 0.31	2.89 ± 0.36	1.25 ± 0.21***
LDL-C, mmol/L	3.22 ± 0.56	3.25 ± 0.37	3.14 ± 0.30	2.57 ± 0.33**
HDL-C, mmol/L	1.48 ± 0.21	1.40 ± 0.18	1.46 ± 0.24	1.80 ± 0.18**

The data are shown as the mean ± S.E.M, **P <0.01 and ***P<0.001 vs start period of the same group.

These data indicated that the lipid metabolism of the participants with T2DM was improved by the high-fiber diet, which revealed that the added fermentable carbohydrates could produce clinically lipid metabolic improvements in the treatment group.

### Measurements of serum inflammatory chemokines levels

**Figure 2:**
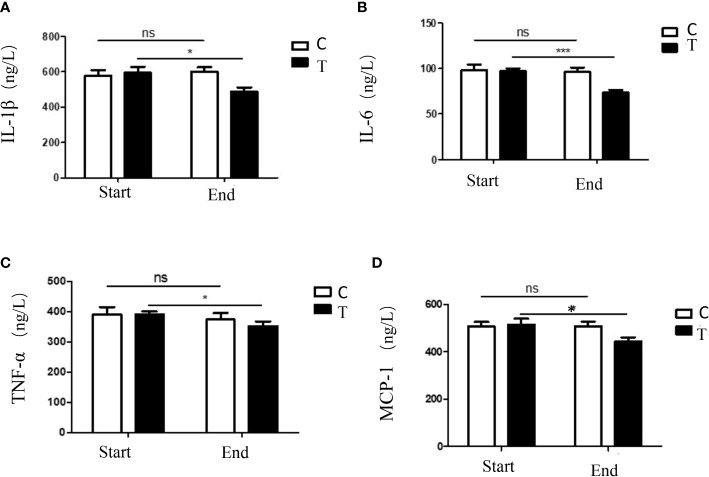
The serum levels of inflammatory chemokines in participants with T2DM altered by the high-fiber diet intake. Expressions of serum levels of IL-1β **(A)**, IL-6 **(B)**, TNF-α **(C)** and MCP-1 **(D)** in participants during the intervention were shown. The symbols * indicates p< 0.05, symbols*** indicates p< 0.001, ns indicates no significant difference.

Serum levels of inflammatory chemokine can be used as indicators of systemic inflammation, in this study, four kinds of serum inflammatory chemokines (IL-1β, IL-6, MCP-1 and TNF-α) in the two groups were measured by ELISA detecting methods. Compared with the control group, the serum levels of inflammatory chemokines in the treatment group were significantly decreased (**Figure 2**). The current results demonstrated that the consumption of a high-fiber diet could decrease the systemic inflammation.

### Evaluations of the depression and anxiety symptom severities

**Figure 3:**
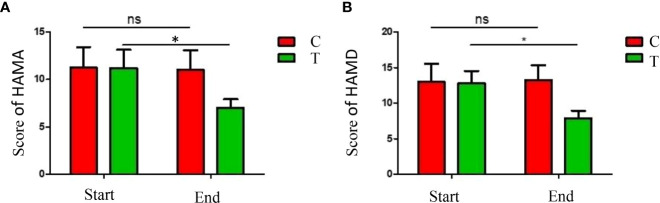
Analysis of the mood symptoms. HAMA scores **(A)** and HAMD **(B)** scores were significantly decreased after the dietary intervention. The symbols * indicates p< 0.05, ns indicates no significant difference.

The depression and anxiety symptom severities were evaluated by HAMA and HAMD questionnaires. The scores of HAMA and HAMD were significantly decreased in patients of the treatment group (*P <*0.05), indicated that the depression and anxiety symptoms were alleviated by the high-fiber diet (**Figure 3**). The results demonstrated that significant improvement in depression and anxiety moods were observed in the treatment group by the uptake of more diverse carbohydrates in the diet.

### Microbial compositional alterations of the gut microbiota

**Figure 4:**
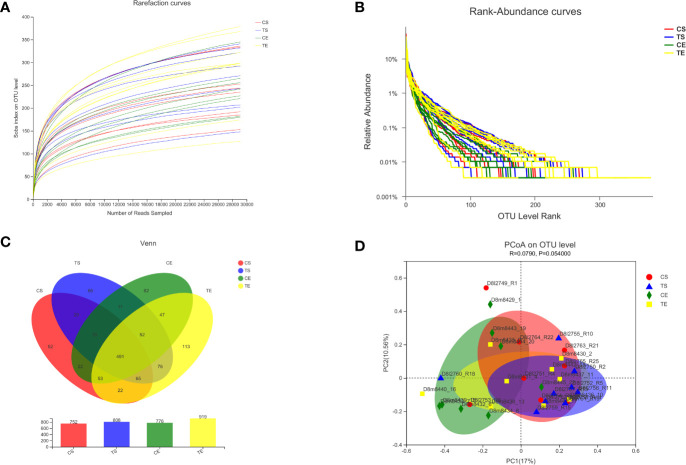
The diversities of gut microbiota before and after the high-fiber diet consumption were analyzed, and the CS and CE groups revealed the start and end phases in the control group, while the TS and TE groups revealed the start and end phases in the treatment group. The rarefaction curves **(A)**, rank abundance curves **(B)**, and Venn diagram **(C)** indicated the alpha diversity of gut microbiota, and the principal coordinate analysis (PCoA) revealed the beta diversity **(D)**.

The raw data obtained from the Illumina MiSeq platform were quality-filtered and demultiplexed to remove invalid and low-quality sequences. A total of 2170797 quality-filtered and chimera-checked sequences were obtained from the 34 samples. The rarefaction curves demonstrated that the sequencing depth was enough for the microbial diversity analysis in the current study (**Figure 4A**). The rank abundance curves revealed that the richness of gut microbiota in the treatment group increased after the high-fiber diet consumption (**Figure 4B**). The Venn diagram indicated that the diversity of gut microbiota in the treatment group was also enhanced by the dietary intervention (**Figure 4C**). The principal coordinate analysis (PCoA) revealed that the different experimental groups were clustered into different communities, which meant that the beta diversity analysis was also changed (**Figure 4D**).

**Figure 5:**
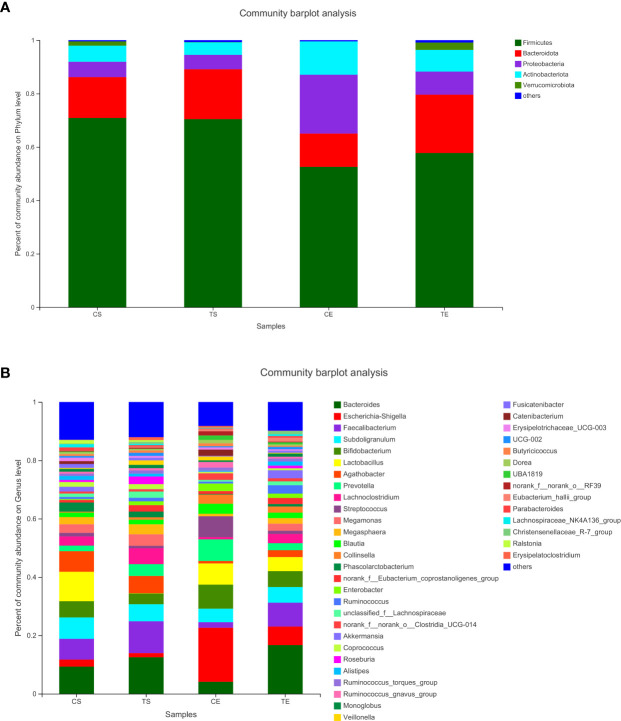
The bacterial communities at the phylum levels **(A)** and the genus levels **(B)**. Less than 1% abundance of the phyla or genra were merged into others.

**Figure 6:**
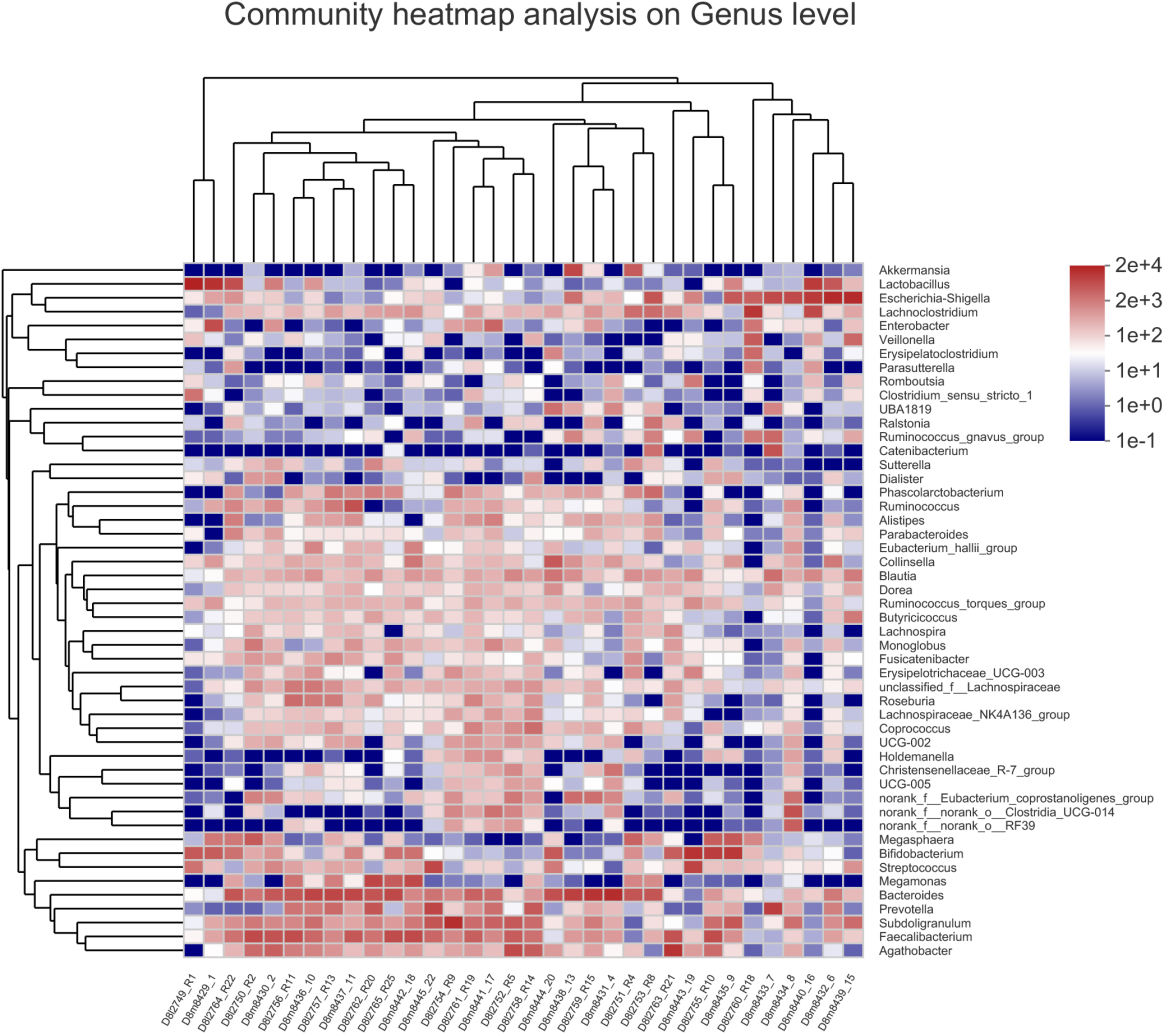
Heatmap of hierarchy cluster results for the abundance of genus in control group and treatment group. The color of the spots corresponded to the normalized and log-transformed relative abundance of the OTUs. The genus names of the OTUs are shown on the right.

**Figure 7:**
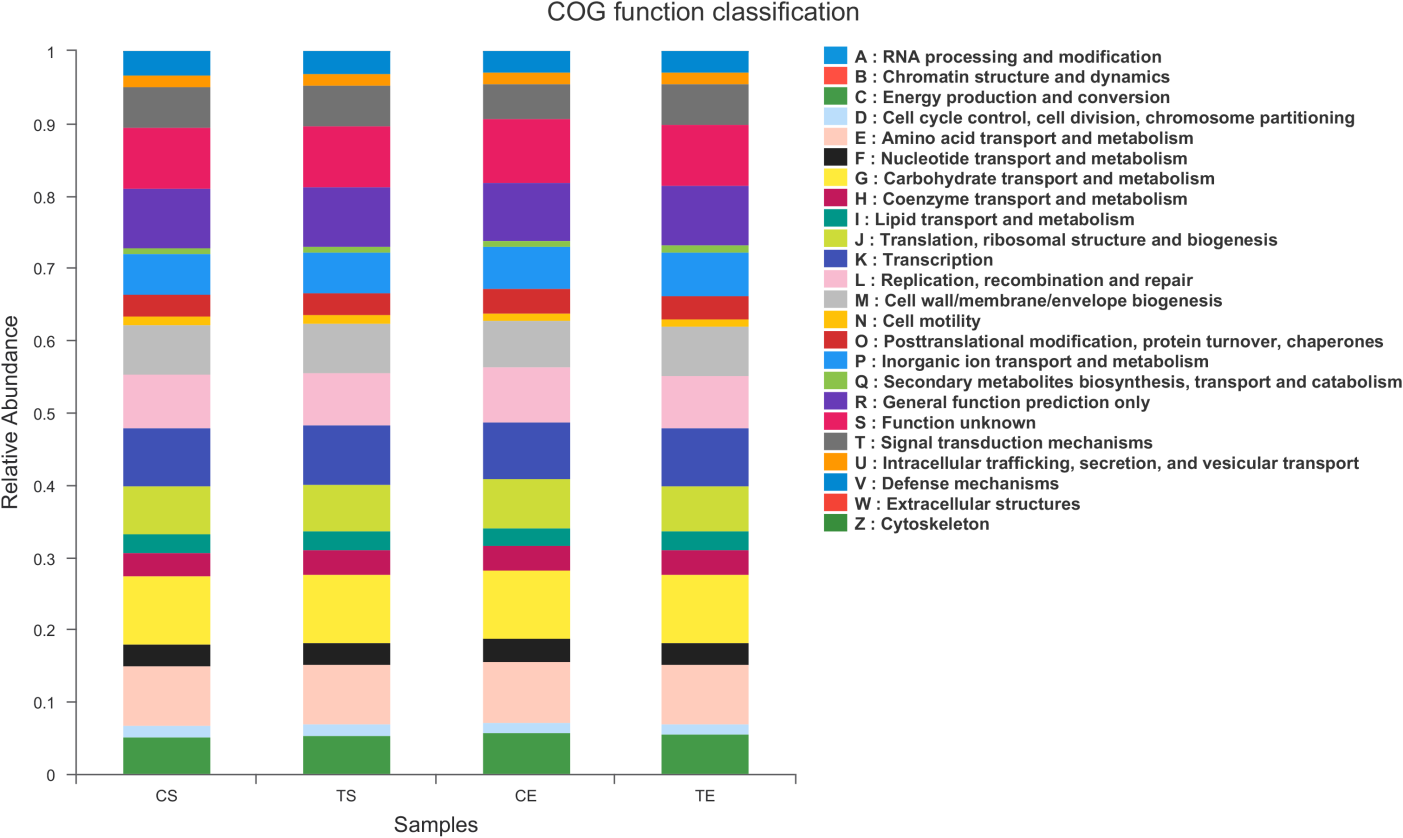
PICRUSt functional prediction was performed using EggNOG database, pathways related to type 2 diabetes were identified and compared.

To assign taxonomic compositions of gut microbiota, the RDP classifier was used to compare the bacterial community structure shifts at the phylum level and genus level, respectively. Taxonomic analysis revealed that a total of 15 phyla, 25 classes, 63 orders, 119 families, 307 genera, and 603 species were identified. At the phylum level, Firmicutes and Bacteroidota account for 79.96% percentages of the total bacterial communities (**Figure 5A**). In the treatment group, the proportion of Firmicutes was decreased while the proportion of Bacteroidota was increased. Therefore, the ratio of Firmicutes to Bacteroidota (F/B value) was significantly decreased in the treatment group (*P*<0.05). Compared with the control group, the composition of Proteobacteria in the treatment group was decreased, indicated that the colonic epithelial oxygenation was altered by the high-fiber diet. In the treatment group, the proportions of several genera beneficial microbes were enhanced, including *Lactobacillus*(0.08% vs. 4.84%)*, Akkermansia* (0.07% vs. 2.74%)*, Bifidobacterium* (3.70% vs. 5.43%)*, Bacteroides* (12.52% vs. 16.67%)*, Ruminococcus* (1.25% vs. 2.88%), and *Blautia* (1.56% vs. 1.87%). However, the relative abundances of *Erysipelatoclostridium* (1.02% vs. 0.09%), *Megamonas* (3.96% vs. 2.43%), *Prevotella* (4.04% vs. 2.40%), *Klebsiella* (0.65% vs. 0.25%), *Desulfovibrio* (0.17% vs. 0.07%)and other opportunistic pathogenswere decreased in the treatment group (**Figures 5B**, **6**). The changes in bacterial abundances at the genus level revealed that the microbial communities were obviously improved by the high-fiber diet. The predicted functions were calculated based on the PICRUSt software, and a total of 22 pathways were predicted in EggNOG (evolutionary genealogy of genes: Non-supervised Orthologous Groups). As shown in **Figure 7**, microbial genes of Lipid transport and metabolism, Inorganic ion transport and metabolism, Nucleotide transport and metabolism, Coenzyme transport and metabolism, Energy production and conversion, Energy production and conversion, Amino acid transport and metabolism, and Carbohydrate transport and metabolism were decresed in the treatment group. However, the microbial genes of Secondary metabolites biosynthesis and transport and catabolism were increased. The predicted pathway changes indicated that carbohydrate and energy metabolism were influenced by the high-fiber diet.

## Discussion

Previous studies had already proved that the gut microbiota was a critical environmental factor for treating T2DM and other metabolic disorders (Liu et al., 2017; C. Zhang et al., 2010). A nutritional diet composed of a high amount of dietary fibers could provide enough carbohydrates for the gut microbiota to ferment, and the microbial metabolites could provide energy supply and regulate the immune function of the host (Justin et al., 2017; Gowd et al., 2019). For example, the SCFAs produced by gut microbiota demonstrated obvious T2DM-alleviating effects (Dalile et al., 2019). In this study, a specially designed high-fiber diet was applied to regulate the composition of intestinal flora in patients with T2DM.

Zhao et al. had revealed that the gut microbiota could bring additional health benefits for patients with T2DM through carbohydrate fermentation and SCFA production (Zhao et al., 2018). Consistent with the previous study, our experiment results also proved that the high-fiber diet could improve the glucose homeostasis of participants with T2DM. When compared with the control group, the serum levels of HbA1c levels and FBG decreased significantly in the treatment group, while the serum levels of insulin and C-peptides increased significantly in the treatment group (**Figure 1**). Simultaneously, the serum lipid profiles were also improved by the high-fiber diet. The decreased the serum levels of the TC, TG and LDL-C and the increased serum levels of HDL-C in the treatment group indicated the lipid metabolism of participants with T2DM was improved after consumption of a high-fiber diet (**Table 2**). Therefore, the dietary source of fibers demonstrated obvious protective impacts on glucose homeostasis and lipid metabolism of the patients with T2DM.

Manipulation of the gut microbiota by prebiotic administration could improve the glucose and lipid metabolism in obese and diabetic mice, and the low-grade inflammation were also decreased (Everard et al., 2011). Cani et al. demonstrated that prebiotic-treated ob/ob mice exhibited a lower level of plasma lipopolysaccharide (LPS) and inflammatory cytokines, and the decreased inflammatory tone was proved to be associated with a lower intestinal permeability and an improved tight-junction integrity when compared to controls (Cani et al., 2009). Further analysis indicated that the inulin-supplemented diet could restore the leptin related pathways (especially AMPK signaling pathway) of ob/ob mice, which was mediated by gut microbiota changes (Song et al., 2019). In the current study, the serum levels of inflammatory chemokines (IL-1β, IL-6, MCP-1 and TNF-α) in the treatment group were significantly decreased when compared with the control group (**Figure 2**). The changes in systematic inflammation might be related to the immune regulatory functions of fermented products of carbohydrates.

The hippocampal neuronal apoptosis and synaptic structural damage were proved to be associated with the overexpression of proinflammatory cytokines and overactivation of microglia and astrocytes, therefore alterations of intestinal flora could inhibit the immune-inflammatory response and signaling by producing SCFAs (Shao et al., 2023). In this research, evaluations of the depression and anxiety symptoms by HAMA and HAMD questionnaires indicated that the depression and anxiety symptoms were improved in the treatment group (**Figure 3**). Therefore, the improvements in emotional moods were observed when more diverse carbohydrates were consumed and the gut microbiota was altered.

The microbial communities of patients with T2DM were obviously changed after the high-fiber diet administration. The richness and diversity of the bacterial community in the treatment group were both enhanced (**Figure 4**). The phylum of Firmicutes and Bacteroidota contributed 79.96% proportion of the gut microbes (**Figure 5A**). Compared with the control group, the composition of Firmicutes increased and the composition of Bacteroidota decreased in the treatment group. Therefore, the decreased ratio of F/B value revealed that the gut microbiota in the treatment group changed obviously at the phylum level. The dysbiotic expansion of anaerobic proteobacteria was considered to have an intimate relation with colonic epithelial oxygenation and intestinal inflammation. When compared with the control group, the composition of proteobacteria decreased obviously in the treatment group. As shown in **Figures 5B**, **6**, several genra of beneficial microbes with lactic acid and SCFAs producing activity were increased after the high-fiber diet intervention, including *Lactobacillus*(0.08% vs. 4.84%), *Akkermansia* (0.07% vs. 2.74%), *Bifidobacterium* (3.70% vs. 5.43%), *Bacteroides* (12.52% vs. 16.67%), *Ruminococcus* (1.25% vs. 2.88%), and *Blautia*(1.56% vs. 1.87%). At the same time, the proportions of *Erysipelatoclostridium* (1.02% vs. 0.09%), *Megamonas* (3.96% vs. 2.43%), *Prevotella* (4.04% vs. 2.40%), *Klebsiella* (0.65% vs. 0.25%), *Desulfovibrio* (0.17% vs. 0.07%), and other opportunistic pathogens were decreased in the treatment group. Therefore, the optimized gut microbiota composition could help the host to modulate immune homeostasis and protect gut barrier function. Moreover, the predicted PICRUSt functions demonstrated that pathways related to the carbohydrate and energy metabolism were influenced by the high-fiber diet (**Figure 7**).

## Conclusions

In the present study, clinical data indicated that the increased availability of fermentable carbohydrates was sufficient to induce metabolic improvements in patients with T2DM. The dietary source of fibers demonstrated protective impacts on the gut ecosystem, and the alteration of the gut microbiota composition improved the glucose homeostasis in patients with T2DM.

## Data availability statement

The datasets presented in this study can be found in online repositories. The names of the repository/repositories and accession number(s) can be found below: https://www.ncbi.nlm.nih.gov/, PRJNA630022.

## Ethics statement

The studies involving human participants were reviewed and approved by Ethics Committee of Shanghai Jiao Tong University School of Medicine. The patients/participants provided their written informed consent to participate in this study.

## Author contributions

Investigation, LC and LR; software, BoL; methodology, HD, CF, CQ, BiL, and RZ; writing—original draft preparation, ZL; review and editing, HL; supervision, ZM. All authors contributed to the article and approved the submitted version.
